# Self-Evaluation Dimensions of Criminal Activity and Prospects Simulation of Persons Serving Custodial Sentence

**DOI:** 10.3390/bs9070075

**Published:** 2019-07-10

**Authors:** Adolfas Juodraitis, Remigijus Bubnys, Odeta Šapelytė

**Affiliations:** 1 Faculty of Health Care, Social Work Department, Šiauliai State College, 76228 Šiauliai, Lithuania; 2 Panevėžys Faculty of Technologies and Business, Technologies and Entrepreneurship Competences Centre, Kaunas University of Technology, 37164 Panevėžys, Lithuania; 3 Institute of Education, Šiauliai University, 76351 Šiauliai, Lithuania

**Keywords:** subjective self-evaluation experience, psychosocial disposition, persons serving custodial sentence, factors of criminal activity, future prospects

## Abstract

The article presents the self-evaluation indicators of the causes of criminal behavior committed by persons serving a custodial sentence at X institution, focusing on imprisonment and modeling of life prospects after release from prison. The main idea of the study is to reveal the subjective self-evaluation experience of persons serving custodial sentence with regard to criminal activity and simulation of future prospects. The scientific discussion of the article presents incarcerated persons’ (N = 58) subjective self-evaluation data collected during the qualitative research and their theoretical-practical interpretation. Convicted persons’ subjective self-evaluation and prospects simulation data enable stating certain features showing the discrepancy between their disposition and achievement of resocialization goals. This confirms the insufficiently interiorized reasonableness of punishment, personal perception, and realized motivation to change.

## 1. Introduction

Trends of interactions of individuals and their groups in different social systems and their manifestation are determined by numerous variables: Dynamics of cognitive and psychosocial development, socio-cultural environment, and change in social stratification, transformations in core social norms, and values, etc. This inevitably expands the range of potential problems in adaptation and socialization processes of the society’s members, interferes with the synergy of the adaptive potential of a certain share of individuals and socialization incentives. One of the consequences of such impact is the problem of discrepancy between social norms and the person’s disposition, which should be related to the trends of behavior tending to violate moral, social, and legal norms in various forms of social interaction.

The etiology of criminal behavior as violation of social norms, which is particularly expressed and dangerous to society, is explained by various, sometimes quite controversial theories that take into account significance and potential danger of the effect of personality traits, subculture, social learning, and other provocative factors and individual segments. Acknowledging the theoretical and practical-applied importance of each of them, it must be noted that a significant condition for the development of the person’s social behavior is the manifestation of the variables of the individual’s disposition. This significantly depends on the specificity of psychosocial development. In this context, we use the term “disposition”, not as one of the constituents of essential trait theories in order to explain the person’s affective, cognitive, and behavioral (“personality-situation”) spheres. “Disposition” is narrowed down to a certain behavioral model in changing psychosocial development conditions (life in freedom–imprisonment institution–simulation of life in freedom) and used to predict the likelihood of change.

It can be assumed that, alongside with a number of other variables, the process of the person’s (un-)successful socialization is also determined by the level of correspondence between perception of fundamental social norms and efforts to follow them. This way, the range of functioning of social norms is inseparable from certain conditions of the individual’s psychosocial development, which could have determined specific interaction between social maturity [1,2,3] and social competence. The said conditions could have also affected different “perception of norms” and “non-standard behaviour”. It is agreed that social competence defines social behavior that is determined by the complex system of social learning, social motives, social skills, and abilities as if ensuring continuity of adequate behavior in various situations.

In rehabilitation and re-socialization processes, attention should be drawn to the important condition: Only upon passing the necessary levels of maturity (of dependence, impulsivity, interpersonal, etc.), the person evaluates the importance of the foundation of moral and legal norms and their essential function regulating behavior [4]. The process of re-socialization could be treated as the return of social status and value by various means to the individual who had lost social trust of the environment, the return of the value of the person as the member of the society, and assistance to him to return to the society rejected by himself [5]. Successful re-socialization requires a complex of elements ranging from the systematic approach, institutional support or legal framework to quality implementation of corrective programs and their continuation. Serving a custodial sentence, grounded on empowerment and motivation rather than oriented to punishment, allowing for control of imprisonment levels and the risk of crime, enables the formation of a set of measures favorable for imprisoned persons’ re-socialization. Such measures include normalization of living conditions in imprisonment institutions, opening of imprisonment, implementation of social climate favorable to convicts, and effective corrective measures [6]. The person’s return to the normal social order requires joint efforts: Both of the person himself and of specialists who are able to empower the person to change.

It is difficult to evaluate how long it can take to achieve appropriate levels, how much effort this change requires, and what additional, or even secondary, variables block the implementation of the intended goals. It is likely that the data of the conducted study, obtained through offenders’ subjective evaluation of certain aspects of their life, will enable identification of a certain part of them.

The scientific and practical relevance of the research presupposes the following problem questions: What factors have determined criminal activity and what consequences are identified by the persons serving custodial sentences? How do persons serving the sentence assess the impact of punishment on personality change at the imprisonment institution? What life goals, change in the meaning of life and priorities do persons serving custodial sentences identify?

The object of the research: self-evaluation dimensions of criminal activity and future prospects of persons serving custodial sentence.

The research aims to reveal the subjective self-evaluation experience of persons serving a custodial sentence with regard to criminal activity and simulation of future prospects.

## 2. Research Methodology

Research place and context. The study was organized in one of the jails located in Lithuania. The purpose and aim of activity of these institutions are to carry out the remand measure—custody, arrest, and imprisonment for persons who have been sentenced to serve their sentence in prison, penitentiary, or open prison colony. The researcher, as a volunteer, took part in the activity of this institution, which enabled him to have a direct possibility to meet research participants. Prior to conducting the research, it was sought to get familiarized with the specificity of such research as thoroughly as possible by analyzing scientific literature [7,8].

In this particular study, it is not aimed to identify the dynamics of dispositions (socio-cognitive approach, personality factors, psychoanalytic approach, etc.) based on the theoretical concept both due to the institution’s rules and to the employed methodology. It is difficult to identify the relationship between subjectivity and objectivity in the respondents’ answers and to accurately name change in the informants’ disposition, in the first place predicting the possible influence of criminogenic factors on successful resocialization.

Research participants. The study involved 58 informants: All men, given actual custodial sentence. Participants were selected employing the purposive-convenience selection method, when the research is conducted in the conditions favorable for the researcher and is directed to identification of common features characteristic to all investigated cases. It is sought to find out how persons given custodial sentence are able to perceive and (self-)evaluate such important segments affecting (un-)successful socialization of persons as giving a sense to the crime and guilt, the personal impact of imprisonment, the attitude towards future prospects, etc. The age of research participants varies from 23 to 64 years (the mean is 38, 5 years), 26 of them have been convicted once; 12, twice; 11, more than twice; and 9, more than ten times.

Research ethics. The study was organized in accordance with the ethical principles of research, which are exceptionally emphasized in the institutions of this type [9,10]. The study was launched upon the receipt of oral consent of the administration representatives of the responsible institution, after familiarizing them with the content of the research instrument and the type of projected dissemination of results. The research was conducted on the basis of the following ethical approaches and principles: The study involved persons who were familiarized with the content of the study in advance, and upon their oral consent, the investigated persons’ participation was voluntary: The participants had the right of personal resolve to take part/not to take part in the study or to withdraw from the study at any stage of the survey. The provided information on the aims of the conducted study was accurate and complete. During the research, legislation, and conventions for the protection of human rights were followed, investigated persons’ privacy and confidentiality were not violated.

Research methods. The data were collected conducting the written survey, employing an open-ended questionnaire. The structure of the research instrument consisted of the following diagnostic areas: Incentives for the criminal activity, the impact of incarceration on the personality, prioritization of life values, and prospects of further life.

The qualitative data analysis was performed employing the qualitative content analysis method [11,12]. Content analysis method for data analysis was determined by several reasons: (1) The previous scientific experience of researchers using this data analysis method [13,14,15,16,17,18]; (2) the content of the text is analyzed consistently and systematically in order to identify the relationships between texts, meanings, qualities, or general themes (in our case dimensions and categories) in the qualitative content analysis, also to analyze them in the context of selected research field. The objective of qualitative content analysis is to systematize and summarize research material, to describe phenomena, to reveal the meanings people give to these phenomena [19,20]. This objective was essential to analyze the diversity of perceptions, behavior, strategies, and interactions among persons serving custodial sentences. (3) An inductive approach was employed to describe little analyzed phenomenon without sufficient knowledge and information seeking to move from the cases of individual, isolated experiences (features, attributes, etc.) towards generalization—common knowledge and conclusions [11,12,21]—in a particular case, towards the development and presentation of latent structure of the experience.

The qualitative content analysis as a method of cognizing the social environment enables the researcher to explain and interpret text data by systematically classifying them. For that purpose, data arrays are identified and coded, qualitative categories are distinguished. The whole process of the qualitative content analysis took place in three stages: preparation, organization and presentation, following the sequence of procedure inherent to the qualitative content analysis: (1) Reading of answers and submerging into the research data, (2) distinguishing of notional units of the analysis, (3) open data coding and abstraction, (4) creation and assignment of subcategories and categories, (5) formulation of topics and grouping of results, and (6) data interpretation and preparation of the report.

## 3. Results

The custodial sentence separates the offender from various functional forms of social systems (work and social activities, family and the circle of close people, ways of spending leisure time, etc.) for various time periods. Naturally, violation of social norms results in respective punishment, which, as the most severe sanction, must lead to corresponding change in the self-evaluation of the offender’s conduct. It must also promote the transformation of personal characteristics and adaptive behavior. Paradoxically, in many cases, and this is confirmed by research conducted by criminologists, sociologists, and psychologists [22,23,24,25,26,27], the custodial sentence often fails to modify behaviour in the penitentiary period by mastering control of socially important traits (self-control, responsibility, social participation, etc.). Furthermore, the more coercive and punitive prisons and their regime, the more violent prisoners might be expected [22]. However, it has to be stated that a share (proportions depend on the individual’s age, education, peculiarities of psychosocial development, frequency of criminal activity, etc.) of convicts treat punishment as the revenge of the society. They interpret it as a stigmatizing action restricting their rights. This disturbs their striving to re-socialize and live full-fledged social life in freedom in the future (convictions, insufficient education and vocational training, family status, etc.). Quite often, such a philosophy of the discrepancy between punishment and imaginary revenge is a peculiar form of defense for convicts (may also be as a component of subculture). It transfers a certain part of responsibility for the crime committed to individual social systems (family, friends, traditions in the environment, etc.) or distinguishes various consequences determined by provocative situations. Naturally, a certain share of convicts acknowledge their guilt, self-evaluate the sequence (manifestation) of both main causes and subsequent consequences, identify ongoing change and the possibility to overcome problems upon their release from prison.

The analysis of the responses given by persons serving the sentence at X institution enables evaluation of the influence of only a certain share of factors that have provoked the crime, the level of perceiving incarceration as adequate punishment and simulation of prospects of life in freedom. Complicated identification of objectivity and subjectivity dominance parameters reconfirms the importance of organizational and management drawbacks of convicts’ unsuccessful (re-)socialization as well as the need for change. It is also important to take into account the circumstance that respondents’ subjective self-evaluation process was to some extent influenced by the components of explanation (excuse) and pretext. The relation of their manifestation is significantly related to attribution of causality and responsibility.

Identifying determinants of criminal activity from a relatively broad and often undefined list of confirmatory statements (economic, socio-economic, personality, external impact, social environment, and other causes), four essential groups of factors were distinguished (see Table 1).

Based on informants’ subjective assessment, the factors that determined the criminal activity are to be related to socioeconomic and inner factors determined by personal lifestyle and to the fact of former dependence. The latter group of factors is considered as one of the key groups that have pushed to criminal activity. In the group of personality-attributive factors particular emphasis should be placed on the dominance of the external locus of control, explaining the causes of the current situation by not blaming internal factors, but the ones that have prevailed in the social environment. Consequences determined by hedonistic incentives, provocation, and the abilities to manage the situation, forming the group of cognitive dissonance factors, come to prominence too.

Recognizing that harmony between social maturity and social competence determines both the individual’s ability to make corresponding decisions and follow social behavior norms, it can be assumed that dispositions of persons exhibiting deviant behavior are determined by numerous variables functioning at varying intensity. One of them is the regularity that the already existing deficit of essential social skills continues affecting the qualitative parameters of social network development [3,28,29], determines low level of self-control and self-regulation [30,31,32,33], and complicates the ability to constructively resolve conflict situations. The other possibility cannot also be rejected, i.e., being affected by the disposition of the same modality and intensity, the person not only does not solve but he himself provokes conflicts and situations threatening surrounding people. Such psychosocial disposition determines manifestation of autonomy dominance (expressed individual functioning) and primitiveness of social responsibility perception, respectively, enhancing the individual’s criminal behavior. No doubt, such interaction complicates the impact of re-socialization processes of a larger share of convicts, while changing/during change of “old” behavior models.

The performed content analysis enabled to identify the incarceration situation as a punitive measure transforming and separating the personality (see Table 2).

Three distinguished groups of content elements reflect radical change in the perception of personal and social life, negative, and relatively positive personality change as a manifestation of punishment for performed actions. Getting into the imprisonment institution has the greatest impact on relationships with one’s family. Informants emphasize the dynamics of feelings of difficulty, hatred, loss of self-confidence, trust in other people, in the society. The psychological state forces constantly thinking about one’s family for being unable “to be a support” and “take care of others”. Summarizing the assessments, it should be stated that the system of imprisonment tortures, punishes a person, makes him evil, and distrustful of the society. In some sense, there is a specific dissonance in the perception and effect of meaningfulness of punishment between punished and punishing persons.

The third area of research is to be related to identification of change in life goals and meaning. It is sought to reveal change in priorities of persons serving the sentence with regard to perception, identification of goals and meaning of life before imprisonment and when they had some experience of isolation from the society (see Table 3).

Three indifferently assessed categories were identified, focusing on change in the perception of personal and family life and axiological change, but at the same time, also on strivings for material benefit and hedonistic goals. It is obvious that imprisonment changes the personality itself and at the same time the attitude towards life and perception of its meaning. The family (according to incarcerated persons’ subjective assessment, to be treated as a value) is often perceived as a bridge between the imprisoned person and the society. Maintenance of close relationships with family members creates preconditions for a more positive adaptation after returning from the imprisonment institution and for maintaining the link with the social environment.

In the eyes of imprisoned persons, change in values is more focused on the perception of freedom, the human as value as well as on the search for inner peace and morality. On the other hand, the priorities defined in hedonistic, mercantile ways are also important.

Identification of change in life priorities and goals should be closely related to implicit priorities and goals raised upon returning from the imprisonment institution. On the other hand, the question can be raised to what extent the very imprisoned persons’ identified change in values coincides with and/or diverges from the goals and expectations to be related to life outside the isolated environment. The aspects of simulation of goals and tasks of life in freedom are presented in Table 4. The surveyed persons’ responses created preconditions to identify five groups of factors of goals and priorities of life in freedom: The group of factors of creating personal and family life and welfare; the group of occupational-professional activity objectives; the group of factors of simulating socially acceptable behavior; the group of factors of simulating migration plans; and the group of relatively indifferently assessed factors (see Table 4).

The analysis of the very informants’ identified change in life goals, values and named expectations shows similarities associated with life in freedom. These are certain coincidences in the group of factors of creating personal and family life and welfare and in the group of factors of modelling socially acceptable behavior (cf. Table 3 and Table 4).

The group of relatively indifferently assessed factors is also given prominence. It is noticed that the respondents are not sure about their future and raise certain doubts and feels the sense of indefiniteness. Indifferently assessed factors partly presuppose uncertainty, indefiniteness among incarcerated persons, and on the other hand, partly diverge from the goals raised by re-socialization.

## 4. Discussion

**Figure 1:**
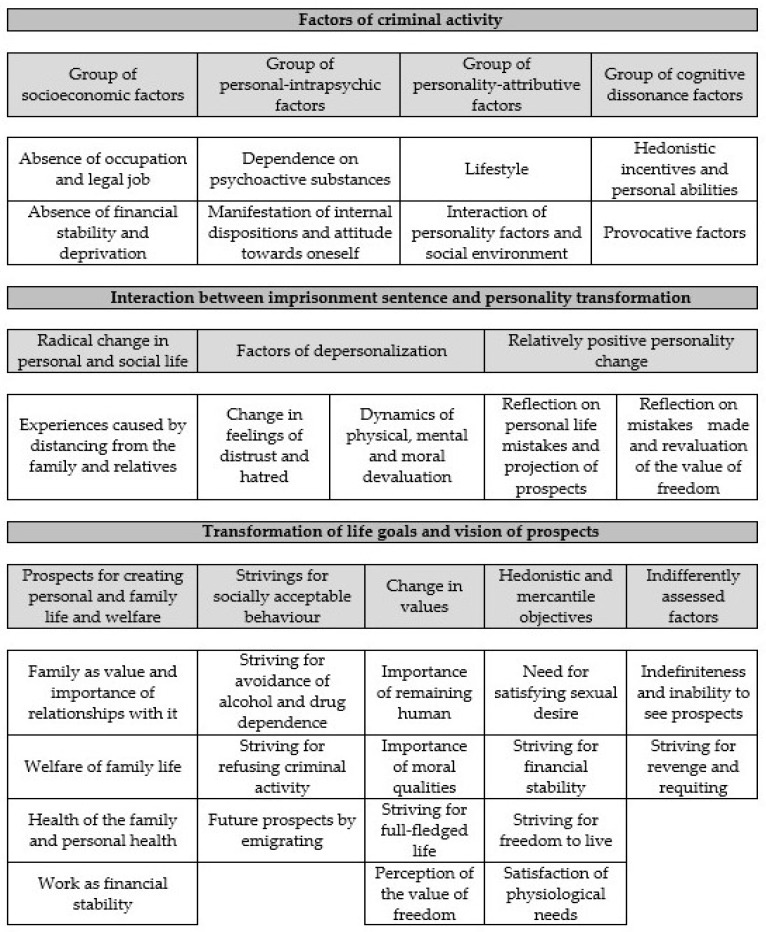
Latent structure of self-evaluation of criminal activity and future prospects of persons serving a custodial sentence.

This study allows for evaluation (possibly subjectively) of only a few possible factors, which have determined the criminal activity and the corresponding custodial sentence (alcohol or drug use, financial status situation, impact of social environment and situations, personality characteristics, etc.), and naturally, often inadequate perception of the punishment imposed. Summarizing the results, the study reveals latent self-evaluation of the criminal activity and future prospects of persons serving custodial sentence (see Figure 1). Authors should discuss the results and how they can be interpreted in the perspective of previous studies and working hypotheses. The findings and their implications should be discussed in the broadest context possible. Future research directions may be highlighted too.

The latent structure of self-evaluation of criminal activity and future prospects of persons serving custodial sentence encompasses three essential dimensions. These should be associated with the factors of criminal activity, the interaction between custodial sentence and personality transformation as well as with the transformation of life goals and vision of prospects. Those data can be linked with others researchers’ works as well [34,35,36], etc. The factors that have determined criminal activity are identified based on subjective self-evaluation from a quite broad and often undefined register of confirmatory statements. Emphasis is placed on socio-economic, personality-related reasons, external impact causes, like social environment, cognitive dissonance, etc. However, external factors that have led to the criminal activity of the individuals involved in the research clearly dominate. Meanwhile, the subjective self-evaluation of the imprisonment situation itself as a punitive measure transforming and separating the personality reflects manifestation of radical change in the perception of personal and social life. It also reveals negative and relatively positive personality-related change. Change in the meaning of life and goals raised upon getting into the isolated environment are oriented to perception of meaning of personal and family life and axiological change. These are to be associated with both revaluation of moral values as well as hedonistically defined guides. Identified trends of change are partly related to the research field of life goals and priorities in freedom. This includes the goals of creating personal, family life and welfare, simulation of socially acceptable behavior, occupational-professional activity priorities, and migration plans. Indifferently treated factors partially presuppose the uncertainty of imprisoned persons, the sense of indefiniteness about the future. On the other hand, they partly diverge from the goals raised by resocialization.

Unambiguous prediction of unsuccessful socialization, “relapse”, or motivation for social integration and adaptation require evaluation of conditions created by the respective social field and preconditions for socialization. Obviously, the nature and severity of the crime committed, adequacy of punishment, their frequency, and imprisonment conditions have different and quite often contradictory effects on punished persons who perceive the custodial sentence as revenge of the society. No doubt, the contradiction between the “deserved punishment” and “revenge” is one of the problems of changing and changes in the offenders’ psychosocial disposition. The solution of this problem is complicated and it is likely that often unsuccessful. It is more often the case that the complex rehabilitation system at the imprisonment institution (activities of a psychologist and social worker, the possibility to study and participate in work activities, volunteer-led therapeutic-occupational sessions, various courses, and other forms of impact), combined with respective monitoring and estimates do not result in required outcomes. Identification of objectively and subjectively operating factors is basically an empirical activity. Its performance is quite complicated and requires not only special knowledge and competencies, but also additional information, possibly enhancing effective selection of measures of impact. One of such source of information could be a certain variety of anamnesis. It would enable to more accurately evaluate the peculiarities of psychosocial disposition manifestation in different stages of the person’s development and the whole of factors that have caused change.

Analyzing the sequence of informants’ statements (acknowledgment of factors that determined the crime, the effect of punishment and loss, etc.) and simulating specific transformation of psychosocial disposition, attention should be drawn to the need for considering individualization and specificity in the re-socialization process. It is noticed that many incarcerated people lose social, political, and economic rights—experience “civil death” [37], which is otherwise referred to by Wacquant [38] as “social death”. As early as 2006, item 103.2 of the European Prison Rules [39] defined the imperative that as soon as possible upon the arrival of the convict, the prisoner’s personal and social situation must be assessed, the individual work plan must be drawn up and release-orientated training strategy must be developed. Sakalauskas [27] observes that individual plan development is one of the essential preconditions for purposeful integration. Such a plan enables foreseeing the content of serving the sentence for the imprisoned person in advance and is the means for the staff of the institution to methodically use existing possibilities and evaluate the steps of its implementation.

## 5. Conclusions

The scheme of variables distinguished in the obtained data analysis process is not finite and is unquestionable (another research is conducted at the institution). Nevertheless, the evaluation of significance and modality of variables in the process of resocialization as if creates corresponding preconditions to simulate the effect of the ways of impact, and if necessary, to change them, having considered problems of interaction of different variables.

Convicted persons’ subjective self-evaluation and prospects simulation data enable to state certain features showing the discrepancy between their disposition and achievement of resocialization goals. This confirms the insufficiently interiorized reasonableness of punishment, personal perception and realized motivation to change. A share of assessments of causes of criminal behavior and models of prospects of life in freedom revealed a certain opposite among persons serving the sentence. This can be treated as a deficit of social maturity and giving a sense to responsibility, which requires additional work of the institution’s staff, and as dominance of a certain established pattern of behavior, namely determining the reference of asocial disposition. The variables identified and named in the study can lead to repeated criminality and increasingly more complex management and implementation of rehabilitation and resocialization processes.

Sub-culture elements prevailing in the system of prisons and respective order of relationships as well as limited possibilities to apply resocialization measures determine that a significant share of persons serving the sentence are hostile to their isolation from the society. This also explains rigidity of convicts’ social maturity and adaptive potential. Simulation of change in offenders’ psychosocial disposition requires both more versatile research encompassing complex personality development characteristics and structural-psychosocial change in the functioning system of imprisonment institutions.

## Figures and Tables

**Table 1:** Identification of factors that have determined criminal activity and consequences.

Category	Subcategory	Proving Statements
Financial problems and absence of occupation	Limitations of occupation and legal job opportunities	Unemployment, lack of occupation… (I1); … I can’t work legally… (I42)
	Financial problems and deprivation in the family	Lack of money (I28); Shortage of money, problems… (I33); Financial difficulties (I11); Absence of constant income (R41); Lack of money, bailiffs take away as much as half of the salary and nothing is left for living (I42); … of course financial shortages, deprivation in the family (I56)
Consequences of dependence	Consequences of substance abuse and dependence	Alcohol (I1); (I6); (I4); (I7); (I14); (I15); (I25); (I26); (I28); (I31); (I32); (I36); … alcohol use had a big influence (I45); It was determined only by alcohol and that’s it (I46); Most often criminal activity was determined by alcohol (I56)
		Drug dependence (I21); Drugs… (I26); Drug withdrawal (I44); My criminal activity was caused by the use of drugs (I50)
Factors of the personality and social environment	Factors of lifestyle	The way… (I18); and the way of life in the beginning… (I20); Immoral life… (I4); Absence of a specific goal of life (I52); I became disappointed with life… (I50)
	Consequences determined by external environment	… inappropriate friends (I6); Friends’ actions (I40); A bad company (I22); Inappropriate company, “fool type acquaintances” (I27); … communication circle… (I28); inappropriate company… (I35); Friends (I49); inappropriate friends (I56); Actions of other people (I17); I got acquainted professedly with a friend and here, drugs, thefts drowned me to hell… (I50)
		People liars, who are saving themselves and write off their crimes on others (I16); I cannot state that the influence of others, because I was not driven by violence. But otherwise, I think, it’s the environment (I9)
	Consequences determined by inner qualities and self-esteem	My character… pride of myself (I2); I was disappointed… and in myself (I50)
		I think the criminal activity was caused by my excessive trust in people, due to which I was engaged, and later I was threatened so that I continue that activity (I53)
		Lack of responsibility… (I45); being under the influence, we behaved completely irresponsibly (I48)
	Consequences of abilities to manage and predict the situation	Desire to easily become rich (I5); … inability to resist easy money (I49)
		… no thinking about possible consequences (I8)
		… inability to evaluate and manage the conflict situation (I58)
	Consequences of provocative factors	Permission to provoke oneself (I58); … provocation of the aggrieved person (I3); … I submitted to provocation (I7); Provocation… (I24)

**Table 2:** Identification of incarceration as a punitive measure transforming and separating the personality.

Category	Subcategory	Proving Statements
Group of factors of perceiving radical change in personal life	Constant experiencing of the feeling of loss of close people and lack of close relationships	Distances from the family (I2); It’s hard to be without family members… (I5); … what affects me most is the lack of family (I9); Personally me, it affects even very strongly, affects psychologically, I am constantly thinking about the family (I11)
		Psychologically this affected very strongly because I can’t communicate with my family, take care of others, be a support for my spouse, I can’t raise our daughter together (I53)
Group of factors determining personality change	Loss of confidence in oneself and the environment	Very bad, you become nervous, lose trust in people (I3); Of course, bad, I became nervous… (I33); This makes me personally nervous (I46)
		In most cases, an imprisoned person loses self-confidence and goes towards destruction (I9); This is ruining me, doesn’t give self-confidence… (I22)
		Negatively, and raises hatred and distrust in people inside (I21); The system of imprisonment tortures, punishes a person, makes him evil, distrustful of the society (I57)
		I was forced to give thought to a lot of things, to think over a lot what I was doing wrong. It ruins, because sometimes they imprison without finding out all the truth, therefore, cause the feeling of distrust in justice, but at the same time, having gone through this, the person strengthens, others break, you need to have a lot of will to pass this road (I4)
	Change in physical, mental and moral aspects of the personality	It badly affects mentally and morally and slightly physically (I27); Not to mention the bodily illnesses, his inner world is ruined. Psyche gets warped (I48); Psychologically, physically, spiritually ruins while you are under arrest (I49)
		The mental state worsened, you become more aggressive (I41) Ruins you–turns you into a fool (I32); Ruins, tortures psychologically (I40); Imprisonment ruins me, I don’t feel stable (I50)
		Imprisonment destroys a person both psychologically and physically, in such conditions diminishes dignity, tortures a person (I16); Imprisonment affects me mentally and morally (I35); Conditions traumatize (I24)
Group of factors of relatively positively assessed personality change	Rethinking of personal life	Allow to give thought how and where you live (I1); Allow to understand further life (I6)
		There is a lot to think about when you temporarily lose your close people, even family, children. I try not to get stressed, I put good ideas for the future (I7)
	Dynamics of reflection on mistakes made and revaluation of the value of freedom	Partially it affects in a good way because there is a lot of time to reflect on the bad things I have done and whether they will allow to correct them (I14); Give time to think about the mistakes made (I52)
		Imprisonment allows to understand that freedom is priceless. Also gives you the opportunity to read many interesting books, because there is no time for that in freedom (I13)
		Makes you give thought, value life differently, helps to understand what freedom really is and what its price is (I45)
		When you appear here, you think over what the person loses, that people don’t value freedom, because one reckless action and you give yourself and your family (I8)

**Table 3:** Identification of change in goals and meaning of life.

Category	Subcategory	Proving Statements
Perception of the meaning of personal and family life	Emphasis on the importance of the family and relationship with them	The most important is family (I2); (I4); (I5); (I6); (I8); (I14); (I15); (I19); (I20); (I24); (I25); (I26); (I29); (I36); (I39); (I44); (I49); For me in life the most important is family (I11); (I12); (I53) (I55); In my life, my family is the most important and it will always be so (I16); My children–my family, relationships with them (I21)
	Exaltation of the family as an essential personal value	The most important thing in my life is my family, and without the family I would be nothing! Family is your people who you trust, when it is difficult for you, they will always help you, you can lean on them, they support you whatever you are! And of course, there would be no love—there would be no love in the world (I56); … to stick together, a harmonious family, to be together again (I7)
		Family, life, children, etc. (I34); Family, relatives, friends… (I35); Family, relatives… (I57); Family, children, relatives (I58); close people (I45); The most important thing for me is the family, my girlfriend and our children (I46); Family members, parents, brothers and all others (I30); The most important thing is family and children (I33); Family, parents (I41); Family, children, wife and all loving me (I9)
	Emphasis on health of the family and personal health	… health (I20); Health of the family (I27); Most importantly, so that there is health (I28); … health of the family (I37); (I35) (I29);
Group of factors of change in values	Importance of remaining human	The most important thing is to remain human (I13); and to remain human (I4); The most important thing is to be human (I23);
	Value of freedom	The most important is freedom (I38); (I40); (I47); (I18); What I didn’t value before is freedom… (I4)
	Full-fledged life	Life itself!!! (I22); To live a complete, normal life (I3)
	Emphasis on understanding, peacefulness and love	… inner peace (I44); … love… (I47); mutual understanding (I57)
	The importance of moral qualities	One’s dignity, honour, honesty, emotional state, the ability to communicate, responsibility, the ability to think positively (I49)
Life objectives grounded on material benefit and pleasure	The need for a woman	Beloved woman (I31); I want to have a lot of women (I32)
	Striving for financial stability	Most importantly money (I42); … money (I47); financial stability (I44)
	Striving for freedom to live	To live in freedom, not only in Lithuania (I54)
	Importance of fulfilling physiological needs	The most important thing is not to be nervous and eat well (I43); To eat, do something, also listen to some radio (I52)

**Table 4:** Simulation of goals and priorities of life in freedom.

Category	Subcategory	Proving Statements
Group of factors of creating personal and family life and welfare	(Re-)creation of family life and striving for its welfare	… to create life, to create a family (I35); To create a family (I2); To get out of here, create a family… (I4); to create a family (I39); With family and relatives (I26)
		Be with one’s family every second, because only when you are imprisoned you can understand that every moment is important and so precious (I53); To start standing up on one’s feet upon return to the family […] and do everything for the sake of the family and live a beautiful life (I9)
		To live family life (I6); Plans upon going to freedom […] to create a successful family further (I7); to take care of the family (I8); And to spend as much time as possible with the family (I5); Calm life with the family (I25)
		To live peacefully with my beloved woman (I31)
		… to live morally and never separate from children (I33); To take care and enjoy children… (I58); To have a nice family and many kids (I18)
Group of occupational-professional activity objectives	Striving for work as financial stability of the family	… to find a job […] to provide a family (I4); To stand up firmly on one’s feet, find a good job so that the family is not short of anything (I11); To work as much as possible and earn… (I5)
	Professional activity as striving for the opportunity of career and adaptation	… I would like to find a job (I7); to restore and continue working activities (I58); to continue working… (I38) Upon release from prison I plan to start working… (I33) I plan to continue working and make a career (I35); After leaving to go to work […] to adapt first of all (I8)
Group of factors of simulating socially acceptable behaviour	Striving for avoidance of alcohol and drug dependence	not to abuse alcohol… (I7); Not to abuse alcohol and stop making such crimes (I46); to attend alcoholics anonymous meetings and to try to live morally (I4); I plan to stop wasting time and to look at life only soberly (I19); Rehabilitation–to stop using drugs… (I44)
	Striving for a full-fledged life by refusing criminal activity	Full-fledged life as I lived (I24); To live a decent life (I41)
		To start everything anew, forget the past, change life comprehensively (I45); To serve the sentence I was given and stop getting entangled in crimes (I30); … no longer make such crimes (I46)
Group of factors of simulating migration plans	Striving for future prospects by emigrating	I plan to live my further life with my family, but not in Lithuania of course (I16); To work, build a better life, create a family, leave Lithuania (I49)
		To emigrate (R54); … foreign countries–work, to create a family. Everything after release, of course (R44)
		To depart from Lithuania, at the same time I’ll change my environment and the circle of friends (I50)
		My plans are really realistic. Upon release, I plan to go abroad to my mother. She has been waiting for me long and I plan to find a job there and be a good person. Create my own family, buy my own dwelling and be happy, not to return to places of imprisonment (I56)
Group of factors of difficult-to-identify life prospects	Dominance of indefinite life prospects	All plans fell apart (I15); I don’t know myself (I23); First you need to become free, and further, we will see (I43); I don’t plan, everything will depend on circumstances (I3)
		Since I am already 64, there is no need, aim or sense of planning anything in further life. And as to the next 10 years that I’ll have to spend in the imprisonment institution, there can’t be any thought about any planning. I’ll have to follow a completely alien, forced but probably necessary regime. There is no use of speaking that somebody will re-educate me, put me on the “right way” or change my character somehow otherwise. Although I am of retirement age, I may be able to do some work. Maybe I will attend some courses. But it’s just from curiosity, not necessity (I48)
		The less you plan your life, the less stress you experience (I51)
	Striving for revenge and requiting	To requite everyone for what they have deserved. Despite anything!!! (I52)
		I will serve a 20-year sentence. When I’m released, I’ll beat somebody to death with a hammer and come back to die in prison (I47)
